# Bioinformatics in Malaysia: Hope, Initiative, Effort, Reality, and Challenges

**DOI:** 10.1371/journal.pcbi.1000457

**Published:** 2009-08-28

**Authors:** Azura M. H. Zeti, Mohd S. Shamsir, Khairina Tajul-Arifin, Amir F. Merican, Rahmah Mohamed, Sheila Nathan, Nor Muhammad Mahadi, Suhaimi Napis, Tin W. Tan

**Affiliations:** 1 Institute of Systems Biology (INBIOSIS), Universiti Kebangsaan Malaysia, Bangi, Selangor, Malaysia; 2 School of Biosciences & Biotechnology, Faculty of Science and Technology, Universiti Kebangsaan Malaysia, Bangi, Selangor, Malaysia; 3 Bioinformatics Research Laboratory, Faculty of Biosciences & Bioengineering, Universiti Teknologi Malaysia, Skudai, Johor, Malaysia; 4 Institute of Biological Sciences, Universiti Malaya, Kuala Lumpur, Kuala Lumpur, Malaysia; 5 Malaysia Genome Institute, Universiti Kebangsann Malaysia, Bangi, Selangor, Malaysia; 6 Department of Cell and Molecular Biology, Faculty of Biotechnology and Biomolecular Sciences, Universiti Putra Malaysia, Serdang, Selangor, Malaysia; 7 Department of Biochemistry, Yong Loo Lin School of Medicine, National University of Singapore, Singapore; University of California San Diego, United States of America

**Box 1**

**Authors' Biography**

Nor Muhammad Mahadi was formerly Professor of Microbiology at Universiti Kebangsaan Malaysia (UKM) and is currently Director General of Malaysia Genome Institute (MGI); Rahmah Mohamed is Professor of Molecular Biology and currently Deputy Vice Chancellor of UKM; and Sheila Nathan is Professor of Molecular Biology at the same university and currently Director of Comparative Genomics and Genetics at Malaysia Genome Institute. The trio pioneered the bioinformatics research initiative in Malaysia and collaborated with Tan Tin Wee in the early development of Asia-Pacific Bioinformatics Network (APBioNet). Tan Tin Wee is now an elected Board Director of the International Society for Computational Biology (ISCB), having pioneered the Internet in Asia and co-founded the Bioinformatics Centre (NUS), Singapore Advanced Research and Education Network (SINGAREN), Asia Pacific Advanced Network (APAN), and APBioNet in the 1990s. Amir Feisal Merican is Associate Professor at Universiti Malaya, Director of the Centre for Computational and Systems Biology at MGI, and the coordinator for the National Bioinformatics Roadmap. Suhaimi Napis is Associate Professor at Universiti Putra Malaysia who is involved in setting up the Malaysia Research and Education Network (MYREN), which now serves as a platform for high-speed network for national bioinformatics research. Zeti Hussein, Mohd Shahir Shamsir, and Khairina Tajul-Arifin are the first batch of government-sponsored Malaysian scientists who have received PhDs in computational biology and bioinformatics from Edinburgh, Exeter, and Queensland, respectively. Zeti is a senior lecturer at UKM and her research focuses on structural bioinformatics and computational systems biology; Shahir is a senior lecturer at the Universiti Teknologi Malaysia and his research focuses on molecular dynamics and biodiversity databases; and Khairina is a lecturer at UKM and her research focuses in protein domain functions and intra-domain functional relationships.

## Introduction

The published articles in *PLoS Computational Biology* on the development of computational biology research in Mexico [1], Brazil [2], Cuba [3], Costa Rica [4], and Thailand [5] have inspired us to report on the development of bioinformatics activities in Malaysia. Rapid progress in molecular biology research and biotechnology in Malaysia has created sufficient demand for bioinformatics in Malaysia. Although bioinformatics in Malaysia started in the early 1990s, the initial focus on the development of the biotechnology industry has curtailed the early gains and overshadowed the systematic development of bioinformatics in Malaysia, which currently lacks in human capital development, research, and commercialization. However, government initiatives have been devised to develop the necessary national bioinformatics network and human resource development programs and to provide the necessary infrastructure, connectivity, and resources for bioinformatics. Stakeholders are experiencing reorientation and consolidating existing strengths to align with the global trends in bioinformatics. This exercise is expected to reinvigorate the bioinformatics industry in Malaysia. Tapping into niche expertise and resources such as biodiversity and coupling it with the existing biotechnology infrastructure will help to create sustainable development momentum for the future. An initiative arose from several senior scientists across local universities in Malaysia to promote this new scientific discipline in the country.

## Early Development

Bioinformatics initiatives in Malaysia began in the 1990s through individual initiatives within academia, offering introductory-level computational biology modules in seminars and workshops. The creation of a National Biotechnology Directorate in May 1995 and the launch of the Multimedia Super Corridor by the government generated a sufficient foundation for bioinformatics to take root in the scientific community. In 1998, Malaysia became one of the founding members of APBioNet (Asia Pacific Bioinformatics Network) [6]. A year later, Malaysia's own network, called the National Biotechnology and Bioinformatics Network (NBBNet), was officially launched [7]. Expansion of NBBNet occurred through the participation of Universiti Putra Malaysia (UPM) via the Bio-Mirror project [8], a collaborative service with IUBio Archive at Indiana University for the distribution of public sequence and bioinformatics data via the collaboration between APBioNet and the Asia Pacific Advanced Network (APAN) [8]. In the 1990s, two centers of excellence were established: the CGAT (Centre for Gene Analysis and Technology) at Universiti Kebangsaan Malaysia (UKM) and the Malaysian Synergy Centre for Biology and Information Technology (MSC-BIT) at UPM. MSC-BIT was given the exclusive right to use and distribute a bioinformatics service called WebANGIS (Australian National Genomics Information Service) developed by the Australian Genomic Information Centre based at the University of Sydney, Australia. MSC-BIT established a Malaysian Node for WebANGIS (MANGIS) to facilitate a nationwide network and information technology infrastructure in support of biotechnology and bioinformatics research in Malaysia. EMASGRID, an NBBNet Grid initiative for Bioinformatics & Computational Biology, was established between UKM, Universiti Sains Malaysia (USM), and Universiti Malaya (UM) [9]. Research in Malaysia saw the commissioning of the country's first Linux Parallel Cluster Computing facility for Bioinformatics at the Malaysia Genome Institute in 2001. Recently, the original NBBNet bioinformatics facility has been upgraded into a Genome Computing Centre to cater to the present and future genome sequencing projects and to support the increasing need in structural bioinformatics. With additional funding approved by the Ministry of Science, Technology and Innovation (MOSTI) toward the end of last year, further upgrading will be carried out this year.

## Integration into National Strategies

Early government initiatives were centered on developing a strong biotechnology core with bioinformatics playing a supporting role [10]. Clusters of technology parks were deemed the best launch strategy. These clusters were intended to comprise biotechnology research institutions, universities, and specialized companies, and would foster the convergence of intellectual expertise and entrepreneurship [11]. Establishing a clustered environment would be an excellent platform for generating bioinformatics research. However, it failed to reach fruition [12], reinforcing the complexity involved in creating successful technology parks [13]. Around the same time, a cluster project called the Multimedia Super Corridor was established to cluster all Information and Communication Technology ICT companies. The government also embarked on the integration of bioinformatics in Malaysia into existing national strategies. Initial recognition of bioinformatics from the industry began when Multimedia Development Corporation (MDC) introduced its Strategic Thrust Area in Research (STAR) program in 2003 with a specific focus in Bioinformatics. The Malaysian MOSTI has also been proactively supporting various scientific and technological research, development, innovation, and commercialization initiatives. Currently, the Malaysian Genome Institute is coordinating the large-scale high-throughput sequencing projects to act as a feeder for a national genomic database and subsequent bioinformatics research analyzing these data. Approximately 20% of the annual budget approved is for bioinformatics-intensive projects and has been utilized to study four selected microbial genomes and transcriptomes: a virus for understanding the evolution of the genome through the sequencing of 1,000 isolates from over a 20-year span; an alkalophilic bacterium for novel biotechnology products; an extremophilic yeast for discovery of novel and efficient enzymes, proteins, and pathways; and a parasite of importance in animal health. Other genome and transcriptome sequencing projects are in the pipeline, including those in plants.

## Human Capital Development

In order to successfully develop the bioinformatics sector in Malaysia, it is imperative that policies and strategies are aligned toward producing and maintaining expert and skilled bioinformatics researchers and knowledge workers. As such, local industry and academic research were in the past affected by the lack of available human capital in the field. While in many countries demand generated by expansion and development of bioinformatics in the biotechnology industry had spurred the creation of many bioinformatics courses [14], the UM undergraduate program offered the undergraduate course in Bioinformatics in November 1999. Initially, these courses were developed by combining computer science elements within a biology course, and this highlighted the pressing need in academia for qualified trainers. By 2003, there was sufficient critical mass for more than 300 participants from Malaysia to attend the 2nd International Conference on Bioinformatics (InCoB) organized by Malaysians in Penang, Malaysia (chaired by author RM). This conference was a remarkable breakthrough at a time when bioinformatics was new to the region, and many participants came to learn about bioinformatics, its scope, and its potential for the first time. Within a short period, several academic institutions in Malaysia have now established academic programs at undergraduate and postgraduate levels specializing in bioinformatics [15]. At the same time, awareness of international e-learning resources, such as the free online bioinformatics training courses coordinated by the S* Alliance, spread to Malaysia [15]. Institutions of Higher Learning (IHLs) that started offering bachelor degrees in Bioinformatics include public universities such as UM, UKM and the Universiti Teknologi Malaysia, as well as private universities, including Management and Science University and Selangor Industrial University. On the postgraduate level, these universities offer only research degrees, with the exception of the UM, which in 2008 began offering a master's degree in bioinformatics through coursework.

## Period of Consolidation

As current bioinformatics research activities approached critical mass, stakeholders in Malaysia began the natural progression of consolidating and preparing for future expansion, and in doing so, the local bioinformatics industry faced some significant challenges. There was a severe lack of skilled bioinformaticians, and the local market was still at a nascent stage. Earlier successful government promotion in biotechnology eclipsed much needed awareness of the potential of bioinformatics, resulting in an absence of awareness of Malaysian biotechnology and bioinformatics companies. This led to insufficient cutting-edge and novel research, which resulted in fewer opportunities for commercialization of products or technologies. This lack of a clear lead agency to drive the growth, development, and advancement of bioinformatics in Malaysia forced MOSTI to call for a workshop to design a National Technology Roadmap for Bioinformatics. This timely call to action was designed to address these challenges and ensure that the development of the field would not be impeded in the coming years. It was also of tremendous value not only for industry, but also academia, research institutions, and government agencies involved in the many activities related to bioinformatics. The framework and roadmap identified potential key thrust areas for research, development, and commercialization (R&D&C) in bioinformatics for Malaysia, as well as the role and impact of bioinformatics on Malaysian stakeholders (researchers, government, end-users, and industry). The technology roadmap for bioinformatics was produced in two stages. The initial roadmap document was produced during the period from 2005 to 2006, spearheaded by the Grid Computing and Bioinformatics Laboratory of MIMOS Bhd (an agency under MOSTI). The document was officially submitted to MOSTI in November 2007. Due to the rapid development of ICT and bioinformatics itself over the past few years, the bioinformatics community felt that a review of the initial document was required, especially to align with current and future global trends in the field. A second workshop was organized by the Division of ICT Policy, MOSTI and the Malaysian Society of Bioinformatics and Computational Biology (MaSBiC) to review the first roadmap in August 2008 and was attended by more than 60 participants representing various segments of the industry. Four key thrust areas in R&D&C were identified: Genomics and Computational Molecular Biology, Structural Bioinformatics, Systems Biology, and Bioinformatics Platforms and Technologies. These areas will be supported by the implementation of (i) the creation of a separate category for bioinformatics under the Malaysian Research and Development Classification System; (ii) the creation of career opportunities; priority for national scholarships, fellowships, and grant schemes; and incentives to attract world class bioinformaticians and technopreneurs; and (iii) the targeted and prioritized “top-down” R&D&C programs of national interest.

## Future Development

### Biodiversity informatics

The future potential of this initiative may lie in the rich biodiversity in Malaysia [16]. Malaysia is one of the top mega-biodiversity countries in the world with a long, established history in its contribution to biodiversity research. However, this legacy is marred by the dispersed distribution of stored biodiversity data in various institutions throughout Malaysia. The current approach involves a two-prong strategy; the first is to maneuver biodiversity information policy and management to make it integral to all national strategy development and decision making regarding the utilization of biological resources. This means considering scientifically sound biological and ecological data as part of the national policy planning process in forestry, agriculture, fisheries, land management, and economic development, and in the regulatory regimes that affect a wide range of human activity. The second approach is to create bridging tools to enable researchers to store, curate, and publish, allowing greater utilization of these tools to biodiversity researchers. Biodiversity data are legacy in nature, usually difficult or even inaccessible, and flat file formatted with the existing geospatial information in numbered coordinates that are almost meaningless in the first instance. Various free Web-based tools have been developed by Universiti Teknologi Malaysia and UKM to allow rapid digitization of legacy biodiversity data (http://birg1.fbb.utm.my/vibigis/). The concerted effort on strategic data management will make biodiversity informatics a suitable platform to harness Malaysia's mega-biodiversity and offer a technological niche within bioinformatics.

### Special incentives

The bioinformatics industry in Malaysia is still young, with only a handful of industry players. Although the number of bioinformatics companies in Malaysia has been growing steadily over the years, special incentives similar to the ones given to the biotechnology sector should be considered. In biotechnology, the Malaysian Government continues to play a key role in attracting investment capital by encouraging commercialization and business management skills, creating best-practice venture capital investment conditions, and building an attractive and internationally competitive business environment. Key incentives available to qualified biotechnology companies with BioNexus status include a 100% tax exemption commencing from the first year the company generates a profit and a five-year Investment Tax Allowance doubling deductions on expenditures on research and development (R&D) and promotion of exports. These fiscal incentives are further strengthened by a set of corporate privileges granted by the Malaysian government including the BioNexus Bill of Guarantees, which guarantees freedom of ownership, unrestricted employment of knowledgeable workers, and freedom to source funds globally [17]. The availability of similar schemes would also boost bioinformatics in Malaysia.

### Quality education

The multidisciplinary nature of bioinformatics has created a niche for specialists trained in both biology and computing and has required a distinct teaching cooperation from experts in these two different areas. Consequently, teaching bioinformatics will require a specialist educator with in-depth knowledge of both of the different components: biology and computer science. Malaysian universities that lack specialist and experienced bioinformatics staff resort to the logical route of interdisciplinary and cross-faculty teaching. However, inter-faculty teaching has subsequently raised the issue of “ownership” and, consequently, concerns over the teaching quality [18]. Owing to the multidisciplinary nature and rapid development of bioinformatics, courses offered by the IHLs must be reviewed and updated on a regular basis. For this reason, a Bioinformatics Education and Evaluation Committee (comprising distinguished academics, government officials, research officers, and industry partners) is proposed to review programs and evaluate quality, establish standards, and serve as a point of engagement with international IHLs or training centers to acquire the latest educational methods and content for bioinformatics education and training. An education roadmap is also essential to develop a bioinformatics education framework at various levels (secondary schools, certificate, diploma, college (undergraduate) degree, and postgraduate degree) in line with the identified key thrust areas in research and development. The committee will also work closely with existing bodies, such as the Ministry of Higher Education Malaysia (MOHE), MOSTI, and the Malaysian Qualifications Agency, to ensure implementation of proper standards in Bioinformatics education and training by the public and private IHLs.

Currently, the quality of bioinformatics education is being improved by investments into capacity building in bioinformatics research, via various governmental research funds from MOSTI and MOHE. Academics are also sent for overseas training that includes postdoctoral training, short courses, and secondary attachments to institutions in Europe, North America, and Asia Pacific.

### Plugging into advanced networks

Malaysia is fortunate to have been one of the early participants of APAN, a network of National Research and Education networks (NRENs) in Asia that connects to the USA Internet2 via the Transpac project and to Europe via the Trans-Eurasia Information Network (TEIN) initiatives. Through such advanced Internet connectivity, unlike other developing countries, most Malaysian bioinformatics initiatives do not face bottlenecks in terms of accessing of biological data, such as genomics and proteomics databases, and bioinformatics portal Web services worldwide. Malaysia's budding bioinformatics community can access the latest software technologies and learn how to build upon them. Malaysia has its own open-source bioinformatics software companies and home-grown bioinformatics companies today, such as Synamatix, which is known for its proprietary core invention, SynaBASE™ and the My Genomics Resource Center (MGRC) platform.

### Scientific activism and the continuing quest for excellence in research

Even as Malaysian bioinformaticians grow their internal strengths in research and education, particularly through the continued scientific activism of its earlier pioneers, it is becoming clear that more of them have extended their scope beyond their initial objectives in research as well as education. Malaysian bioinformaticians have served in key positions in bioinformatics organizations such as APBioNet and in outreach workshops of the Association for Southeast Asian Nations (ASEAN). Their increasing participation in the global arena signals the continued organic growth of bioinformatics within the country. Another key indicator is the growing rate of publications in bioinformatics journals as well as the incorporation of bioinformatics methodology in publications in life science research journals. Compared to ASEAN neighbors, in the resolution of the 1st ASEAN-China Bioinformatics Workshop 2004 in Beijing, carried out in conjunction with APBioNet, Malaysia was classified as an “Advanced Level” nation together with the Philippines, Singapore, Thailand, and Viet Nam in its implementation of bioinformatics, which Malaysia started in the 1990s [19]. Relative to other ASEAN countries with less well developed bioinformatics activities Malaysia has seen exceptional growth. In contrast, in the past five years, China and India have both experienced tremendous progress, with many scientific publications in bioinformatics or bioinformatics-related subjects attributable to the Asian region coming from their institutions (personal communication, Elsevier). Malaysia's progress or, for that matter, that of any other individual ASEAN country, compared to the sheer population of these two highly populous nations is unsurprisingly modest. Whereas Japan has demonstrated its capabilities as a global player in bioinformatics databases, including the hosting of DDBJ and PDBj as part of a global consortium maintaining primary biological data sources, and China and India have both certified EMBnet nodes, none of the ASEAN countries has any certified bioinformatics database mirror resource. There is the Pan-Asia SNP initiative across many countries in our region, but that, or any other initiative in the region, has yet to lead to a stably maintained home-grown data resource for global use. In the context of these major indicators, the challenges facing Malaysia and countries in ASEAN are many. With the world's worst financial crisis yet to be played out, most of our resources will be focused on front-line economic issues, while biotechnology growth will be muted, and bioinformatics demand is likely not to be as robust as before.

## Conclusion

Bioinformatics represents a potentially new growth area for Malaysia to build upon, taking into account the country's strong ICT foundation and its tremendous effort in the biotechnology arena. Initiatives recommended for the advancement of bioinformatics and grid computing will be made with a view to undertaking research to support product and process development for the agriculture, healthcare, and industrial sectors. Bioinformatics is necessary for Malaysia to remain current with the biomedical, biotechnical, and agricultural sectors. Malaysia may have missed the initial entrance into the field, but as the field of bioinformatics matures, the country can develop its own market by avoiding mistakes made by developed countries.
