# Correction: Bioinformatics in Malaysia: Hope, Initiative, Effort, Reality, and Challenges

**DOI:** 10.1371/annotation/064aa20e-b7de-4bc0-a2ef-6e8ee3d4de65

**Published:** 2009-09-22

**Authors:** Azura M. H. Zeti, Mohd S. Shamsir, Khairina Tajul-Arifin, Amir F. Merican, Rahmah Mohamed, Sheila Nathan, Nor Muhammad Mahadi, Suhaimi Napis, Tin W. Tan

The address given for author affiliation number 5 is incorrect. The correct address is Malaysia Genome Institute, Ministry of Science, Technology and Innovation, UKM-MTDC Technology Centre, UKM Bangi Selangor, Bangi, Selangor, Malaysia.

